# Change of the airway space in mandibular prognathism after bimaxillary surgery involving maxillary posterior impaction

**DOI:** 10.1186/s40902-016-0071-3

**Published:** 2016-06-07

**Authors:** Woo-Young Lee, Young-Wook Park, Kwang-Jun Kwon, Seong-Gon Kim

**Affiliations:** Department of Oral and Maxillofacial Surgery, College of Dentistry, Gangneung-Wonju National University, 7 Jukheon-gil, Gangneung, Gangwondo 210-702 South Korea

**Keywords:** Orthognathic surgery, Airway management, Cone-beam computed tomography

## Abstract

**Background:**

The purpose of this retrospective study was to develop a two- and three-dimensional analysis of the airway using cone-beam computed tomography (CBCT) and to determine whether the airway space would be changed in mandibular prognathism after bimaxillary surgery involving maxillary posterior impaction.

**Methods:**

Patients requiring orthognathic surgery from 2012 to 2014 were recruited for this study. CBCT scans were obtained at three points: preoperatively (T0), immediate postoperatively (T1), and after 6 months postoperatively (T2). The nasopharynx, oropharynx, and hypopharynx were measured on the CBCT scan for each patient in a repeatable manner. With the midsagittal plane, linear measurements in the middle of each were obtained. For the CBCT, volumetric measurements of each and total airway were obtained.

**Results:**

A total of 22 consecutive patients (11 men and 11 women) were included in the present study. The total volume was significantly reduced (*p* < .001). However, the change of the diameter and volume of the nasopharynx was not statistically significant (*p* = .160, *p* = .137, respectively). In the oropharynx, the change of both the diameter and volume showed statistical significance between preoperatively and immediate postoperatively (*p* < .001, *p* = .001, respectively) and also preoperatively and after 6 months postoperatively (*p* = .001, *p* = .010, respectively). In the hypopharynx, the change of both the diameter and volume showed statistical significance between preoperatively and immediate postoperatively (*p* = .001, *p* < .001, respectively) and also preoperatively and after 6 months postoperatively (*p* = .001, *p* < .001, respectively).

**Conclusions:**

The bimaxillary surgery involving maxillary posterior impaction can reduce the volume of airway in the patients of mandibular prognathism. Although total airway volume was reduced significantly, the changes in the volume and diameter of the nasopharynx were not statistically significant. The maxillary posterior impaction affects on the nasopharyngeal airway minimally.

## Background

Typically, class III malocclusion patients represent a combination of facial malformations in the skeletal and dental alveolar bone [1]. Most of these patients can exhibit mandibular prognathism and maxillary retrognathism. For the establishment of the patient’s optimal occlusion and profile, it is necessary to perform orthognathic surgery, especially bilateral sagittal split ramus osteotomy (SSRO) or vertical ramus osteotomy (VRO) [2]. However, some patients required both maxillary and mandibular surgery, the so-called bimaxillary orthognathic surgery [3]. Clockwise rotation of the maxilla (posterior impaction) can achieve correction of acute nasolabial angle and stabilization of the occlusal plane.

But this surgical movement of the maxilla may reduce the volume of airway space [4]. In the case of the class III malocclusion patients performed with orthognathic surgery, it can change the position of the hyoid bone and tongue, and the base of the tongue moves to the posterior, which will increase the contact surface between the soft palate and tongue [5, 6]. As a result, the pharyngeal airway space is narrowed [7]. Up to date, there are many reports of airway reduction in mandibular set back surgery but rare in maxillary posterior impaction.

In these patients, cone-beam computed tomography (CBCT) has been attracting attention for the three-dimensional assessment of the airway, an important role in the process of diagnosis in place of the conventional CT [8, 9]. CBCT is equipped with a fast scanning equipment and low exposure to radiation as compared to conventional CT [9, 10]. As it can observe on various directions for evaluation of three-dimensional airway reconstruction, it can be suitable for use as a measuring apparatus for a change in the airway between preoperative and postoperative procedure [11].

The purpose of this retrospective study was to develop a two and three-dimensional analysis of the airway using CBCT and to determine whether the difference in the airway volume would be changed with posterior impaction of maxilla in orthognathic surgery.

## Methods

### Patient analysis

From January 2012 to January 2014, a selected group of 22 patients with skeletal class III malocclusion who have complete records were chosen (Tables 1 and 2). Patients with systemic surgical contraindications were excluded from the study. The operations have been performed by the experienced surgeon, and anesthesia was administered by an experienced anesthetist. After surgery, most patients remained in the hospital for 7 days. A standard regimen of antibiotics was administered for 7 days according to clinical conditions. All patients were admitted 1 day before the surgery. A prophylactic antibiotic treatment (augmentin or cefazolin) was routinely given with the induction of anesthesia. NSAID (ketorolac tromethamine) was given for postoperative analgesia. Corticosteroid was given for 3 days via tapering process. All patients were done with intermaxillary fixation by elastic rubber to fit in occlusal stent. When they would be discharged, intermaxillary fixation was removed, and rubber guiding was done.

**Table 1:** Patients information

Patient no.	Age (years)	Sex	Mx post. impaction^a^ (mm)	Fixed reference point	A-P movement of maxilla (mm)	Mn set back (mm)	Genioplasty (mm)
1	22	M	5	ANS	–	12	–
2	23	M	2.5	ANS	–	11	–
3	19	M	5	ANS	–	13	–
4	20	M	2	ANS	–	9.5	Reduction, 4
5	21	M	3	ANS	–	15	Advancement, 4
6	21	M	6	Incisor tip	Advancement, 1	13	Advancement, 6
7	22	M	3	ANS	–	10	Advancement, 6
8	22	M	4	ANS	–	10.5	Advancement, 4
9	24	M	3.5	ANS	–	9	Reduction, 4
10	20	M	5	ANS	–	9	–
11	23	M	5	ANS	–	14.5	–
12	17	F	2	ANS	–	5	–
13	26	F	5	ANS	–	10	–
14	19	F	3	ANS	–	9	Reduction, 4
15	32	F	5	ANS	–	7.5	–
16	20	F	3.5	ANS	–	12	Advancement, 4
17	20	F	5	ANS	–	8	Advancement, 4
18	19	F	4	ANS	–	15	Advancement, 4
19	20	F	6	ANS	–	7	–
20	18	F	3	ANS	–	13	Reduction, 4
21	23	F	5	ANS	–	6.5	–
22	33	F	5	ANS	–	5.5	–

*Abbreviations*: *Mx* maxillary, *Mn* mandibular, *Post* posterior, *ANS* anterior nasal spine, *A-P* anteroposterior

^a^Maxillary posterior impaction was defined as the upward movement of the posterior nasal spine (PNS) in fixation of reference point

**Table 2:** Sample mean

	Male (*n* = 11)	Female (*n* = 11)	*P* value
Age (SD)	21.54 (1.50)	22.45 (5.53)	.609
Mn. set back (SD)	10.83 (1.91)	9.22 (2.76)	.130
Post. impaction (SD)	3.72 (1.36)	3.77 (1.32)	.938

Mandibular set back and posterior impaction are expressed by millimeters. The *t* test was used, and *P* values <.05 are statistically significant

*Abbreviations*: *Mn.* mandibular, *Post.* posterior, *SD* standard deviation

### Radiographic analysis

The radiologic information was taken by cone-beam computed tomography (Alphard, Asahi Roentgen Co., Kyoto, Japan) which was used in the Gangneung-Wonju National University dental hospital, Department of Oral and Maxillofacial Radiology for patient radiographic evaluation. The subjects were positioned with the Frankfort horizontal plane parallel to the floor and instructed to maintain maximum intercuspation with the tongue touching the palate and to avoid swallowing during the scanning period. The three-dimensional image reconstruction was performed to use of the software (Xelis dental, Infinitt Health Care, Seoul, Korea).

### Evaluation of airway change

CBCT scans were obtained at three points: preoperatively (T0), immediate postoperatively (T1), and after 6 months postoperatively (T2). The nasopharynx, oropharynx, and hypopharynx were measured on the CBCT scan for each patient in a repeatable manner at each point (Table 3.). But in this study, lower margin of nasopharynx and upper margin of oropharynx were changed, because maxillary posterior impaction could change the height of posterior nasal spine (PNS). So, we divided the nasopharynx and oropharynx by the plane parallel to Frankfort horizontal plane (FH) passing through the anterior nasal spine (ANS).

**Table 3:** Anatomical limits of the airway

Region	Limit	Anatomical
Nasopharynx	Anterior	Frontal plane perpendicular to FH passing through PNS
	Posterior	Soft tissue contour of the pharyngeal wall
	Upper	Soft tissue contour of the pharyngeal wall
	Lower	Plane parallel to FH passing through ANS and extended to the posterior wall of the pharynx
Oropharynx	Anterior	Frontal plane perpendicular to FH passing through PNS
	Posterior	Soft tissue contour of the pharyngeal wall
	Upper	Plane parallel to FH passing through ANS and extended to the posterior wall of the pharynx
	Lower	Plane parallel to FH plane passing through C2ai
Hypopharynx	Anterior	Frontal plane perpendicular to FH passing through PNS
	Posterior	Soft tissue contour of the pharyngeal wall
	Upper	Plane parallel to FH plane passing through C2ai
	Lower	Plane parallel to FH connecting the base of the epiglottis to the entrance to the esophagus

*Abbreviations*: *FH* Frankfort horizontal plane, *ANS* anterior nasal spine, *PNS* posterior nasal spine, *C2ai* anteroinferior point of secondary cervical vertebrae

**Fig. 1:**
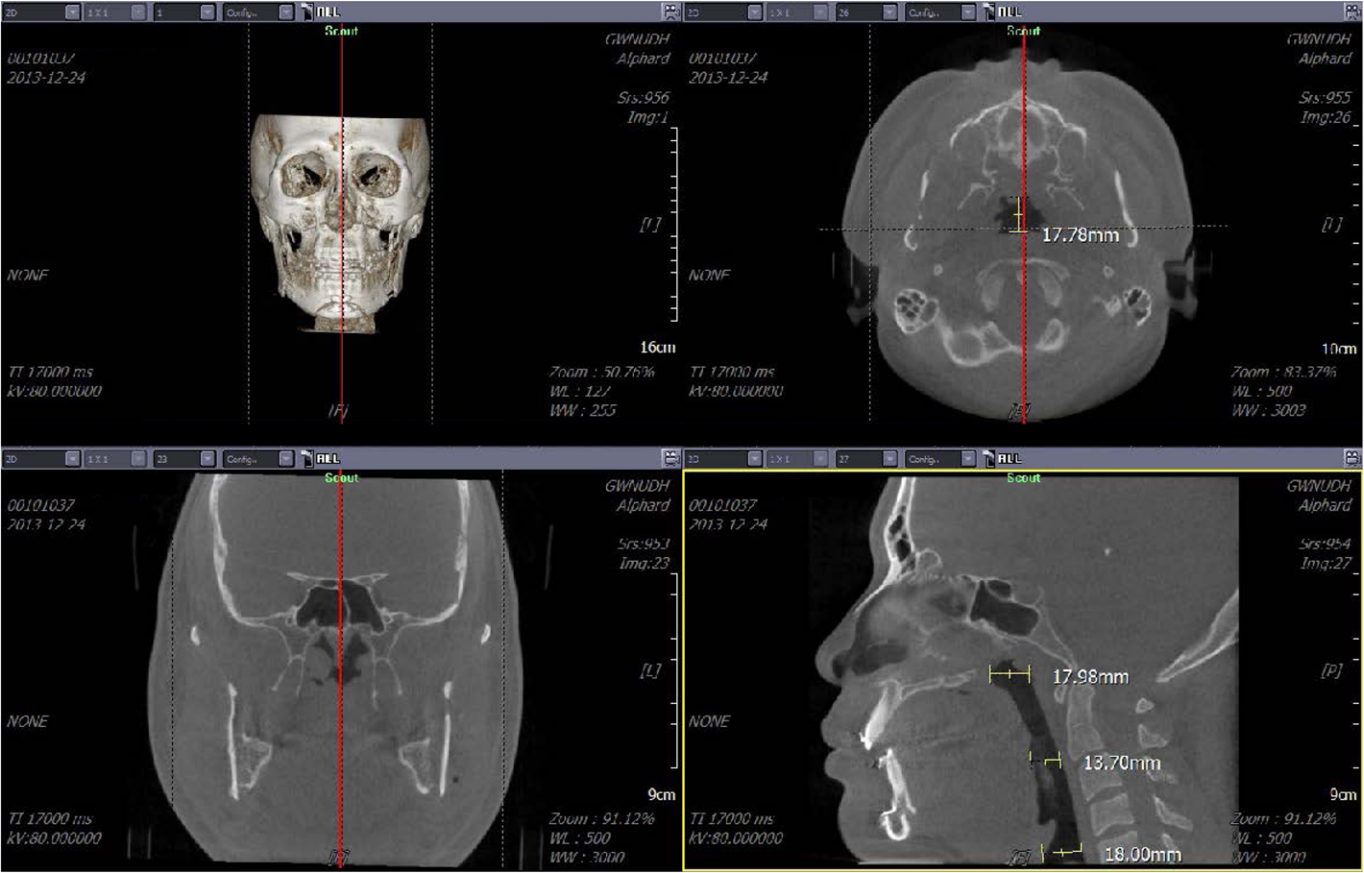
Measuring the length of the airway

**Fig. 2:**
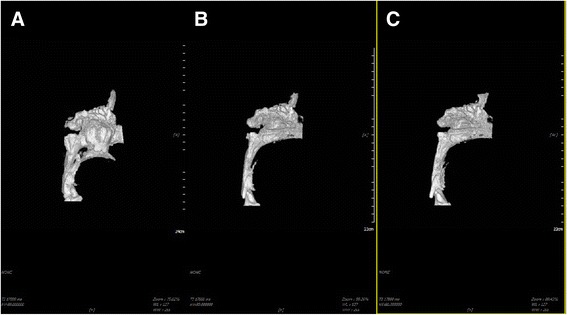
Volumetric analysis of the three-dimensional reconstruction of the airway. **a** Preoperatively. **b** Immediate postoperatively. **c** Six months postoperatively

Three linear measurements and volumetric measurements were made for each image, respectively. Initially, the examiner measured the airway’s anteroposterior length in the midsagittal plane slice at three levels (Fig. 1): anterior nasal spine (the inferior border of nasopharynx), most anteroinferior point of second cervical spine (the inferior border of oropharynx), and the base of epiglottis (the inferior border of hypopharynx). The airway space within the anatomic borders was isolated, and the volume of the each airway was measured after exposing only the airway part (Fig. 2).

### Statistics

The paired *t* test was used for tests of significance in patients preoperatively (T0), immediate postoperatively (T1), and after 6 months postoperatively (T2). Results are expressed as mean ± SD, and statistical significance was accepted at *P* <.05. Statistical analyses were performed with SPSS for Windows, version 19.0 (SPSS Inc., Chicago, USA).

## Results

The patient information data are summarized in Tables 1 and 2. A total of 22 consecutive patients (11 males and 11 females) were included in the present study, with an age range of 17 to 33 years. The average ages of men and women patients were 21.54 and 22.45, respectively. All patients received surgery for posterior impaction of the maxilla and mandibular set back surgery. The average movement of the maxillary posterior impaction is 3.72 mm in male patients (SD = 1.36) and 3.77 mm in female patients (SD = 1.32), and the average movement of mandibular set back is 10.83 mm in male (SD = 1.91) and 9.22 mm in female. There was no significance between male and female.

The linear measurements are summarized in Table 4. The change of diameter in the nasopharynx was not statistically significant between preoperatively and immediate postoperatively (*p* = .160). It did not show a statistically significant difference in immediate postoperatively and after 6 months postoperatively (*p* = .556). Also, there was no statistical significance in the difference between preoperatively and after 6 months postoperatively (*p* = .339). The change of diameter in the oropharynx was statistically significant between preoperatively and immediate postoperatively (*p* < .001). It did not show a statistically significant difference in immediate postoperatively and after 6 months postoperatively (*p* = .253). However, it presented statistically significant in the difference between preoperatively and after 6 months postoperatively (*p* = .001). The change of diameter in the hypopharynx was statistically significant between preoperatively and immediate postoperatively (*p* = .001). It did not show a statistically significant difference in the immediate postoperatively and after 6 months postoperatively (*p* = .932). However, there was statistical significance in the difference between preoperatively and after 6 months postoperatively (*p* = .001).

**Table 4:** Comparison of the linear measurement on preoperatively (T0), immediate postoperatively (T1), and after 6 months postoperatively (T2)

*n* = 22	T0	T1	T2	*P* value		
	Mean (SD) percentage	Mean (SD) percentage	Mean (SD) percentage	T0–T1	T1–T2	T0–T2
Nasopharynx	19.74 (3.77) 100 %	19.15 (3.24) 97.0 %	19.43 (3.89) 98.4 %	.160	.556	.339
Oropharynx	17.54 (5.07) 100 %	13.58 (4.58) 77.4 %	14.33 (4.04) 81.7 %	<.001	.253	.001
Hypopharynx	18.21 (6.17) 100 %	14.86 (4.20) 81.6 %	14.81 (3.94) 81.3 %	.001	.932	.001

Linear measurements are expressed by millimeters. The preoperative state (T0) was defined as 100 %. The paired *t* test was used, and *P* values <.05 are statistically significant

*Abbreviations*: *T0* preoperatively, *T1* immediate postoperatively, *T2* after 6 months postoperatively, *SD* standard deviation

The volumetric measurements are summarized in Table 5. The change of volume in the nasopharynx was not statistically significant between preoperatively and immediate postoperatively (*p* = .137). It did not show a statistically significant difference in immediate postoperatively and after 6 months postoperatively (*p* = .405). Also, there was no statistical significance in the difference between preoperatively and after 6 months postoperatively (*p* = .358). The change of volume in the oropharynx was statistically significant between preoperatively and immediate postoperatively (*p* < .001). It did not show a statistically significant difference in immediate postoperatively and after 6 months postoperatively (*p* = .361). However, it presented statistical significance in the difference between preoperatively and after 6 months postoperatively (*p* < .001). The change of volume in the hypopharynx was statistically significant between preoperatively and immediate postoperatively (*p* = .010). It did not show a statistically significant difference in immediate postoperatively and after 6 months postoperatively (*p* = .836). However, there was statistical significance in the difference between preoperatively and after 6 months postoperatively (*p* = .007). Total volume decreased significantly preoperatively and after 6 months postoperatively compared preoperatively (*p* < .001, both).

**Table 5:** Comparison of the volumetric measurement on preoperatively (T0), immediate postoperatively (T1), and after 6 months postoperatively (T2)

*n* = 22	T0	T1	T2	*P* value		
	Mean (SD) percentage	Mean (SD) percentage	Mean (SD) percentage	T0–T1	T1–T2	T0–T2
Nasopharynx	5363.45 (1895.90) 100 %	5013.05 (1562.34) 93.5 %	5214.30 (1892.05) 97.2 %	.137	.405	.358
Oropharynx	17,833.09 (9130.96) 100 %	10,989.30 (6776.01) 61.6 %	11,860.33 (6187.34) 66.5 %	<.001	.361	<.001
Hypopharynx	7175.34 (5344.89) 100 %	4635.66 (2513.35) 64.6 %	4568.04 (2324.86) 63.7 %	.010	.836	.007
Total	30,371.88 (13,547.62) 100 %	20,638.01 (9585.39) 68.0 %	21,642.67 (9164.16) 71.3 %	<.001	.445	<.001

Volumetric measurements are expressed by cubic millimeters. The preoperative state (T0) was defined as 100 %. The paired *t* test was used, and *P* values <.05 are statistically significant.

*Abbreviations*: *T0* preoperatively, *T1* immediate postoperatively, *T2* after 6 months postoperatively, *SD* standard deviation

Comparisons of the change in total volume between male and female are summarized in Table 6. Female patients showed the narrower airway after surgery compared to male patients. There was significant difference between immediate postoperatively and after 6 months postoperatively (*p* = .021).

**Table 6:** Comparison of the change in total volume between male and female

	T0–T1	*P* value	T1–T2	*P* value	T0–T2	*P* value
	Mean (SD)		Mean (SD)		Mean (SD)	
Male (*n* = 11)	6733.05 (5415.95)	.112	2407.24 (1760.56)	.021	5925.27 (4242.12)	.058
Female (*n* = 11)	13,169.83 (9998.66)		6883.35 (4202.39)		11,646.18 (9638.95)	

Volumetric measurements are expressed by cubic millimeters. The independent *t* test was used, and *P* values <.05 are statistically significant

*Abbreviations*: *T0* preoperatively, *T1* immediate postoperatively, *T2* after 6 months postoperatively, *SD* standard deviation

## Discussion

In the patients of skeletal class III malocclusion who were treated with the surgical procedure, it causes a change in the position of the tongue and hyoid bone and the tongue base moved to the posterior [12, 13]. As a result, the contact surface would increase between the soft palate and the tongue and consequently decrease the pharyngeal airway space [14]. This mechanism brings about morphologic change of oro-pharyngeal area and causes problems such as sleep apnea and snoring [1]. Riley et al. reported that if the pharyngeal airway space is less than 11 mm, the distance from the mandibular plane to the hyoid bone is more than 15 mm, it can cause sleep apnea or snoring [15]. Partinen et al. reported that if the pharyngeal airway space on the tongue base is less than 5 mm and the distance from the mandibular plane to the hyoid bone is more than 24 mm, it can bring about severe respiratory disorder [16].

Evaluation of the airway through the CBCT is reliable. In the airway analysis, many studies have used CBCT than conventional CT [17, 18]. CBCT is equipped with fast scanning equipment and low exposure to radiation as compared to conventional CT [8–10]. Mattos CT et al. reported that CBCT evaluation of the upper airway was reliable. In the study, the authors evaluated linear and volumetric measurements by CBCT [19]. Vizzotto MB et al. reported that both lateral cephalogram and 3D reconstruction by CBCT were suitable for evaluation of airway space [20]. In our study, there were minimal linear difference on midsagittal plane and axial plane. And patient position was reproducible. So, CBCT evaluation of this study was reliable.

In this study, we compared the changes in airway volume and diameter of the patients who underwent maxillary posterior impaction surgery. All patients were accompanied mandibular set back surgery via SSRO or VRO. Although total airway volume was reduced significantly, the changes in volume and diameter of nasopharynx were not statistically significant. The maxillary posterior impaction affected minimally on the nasopharyngeal airway. The average movement of 4 mm is very small so that it could not result in airway change. However, oropharynx and hypopharynx showed statistically significant differences between preoperatively and postoperatively. In the author’s opinion, this was due to mandibular set back movement. The amount of movement of the mandible is usually larger than that of the maxilla. And this mandibular movement is granted a direct effect on the position of the tongue and hyoid bone. Therefore, the diameter and volume of the oropharynx and the hypopharynx are reduced, and total airway volume was also decreased.

Female patients showed the narrower airway after surgery compared to male patients. The reduction of the airway was related not only to the position of the tongue and hyoid bone but also to the edema of the respiratory mucosa. Articles which analyzed the changes in the edema by gender are rare. It was believed that this was the result of the difference in the hormone between the male and female. More research is needed.

**Fig. 3:**
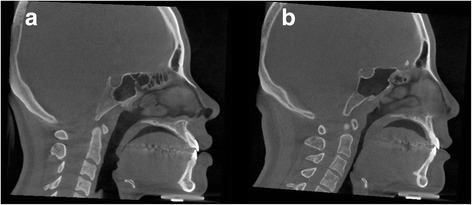
Sagittal plane view after 6 months postoperatively. The airway of patient **b** was narrower than that of **a**. However, patient **a** showed snorting, but not **b**

Snoring occurred in one patient who underwent both SSRO and maxillary posterior impaction. She had narrow airway before the surgery. Comparing to preoperative CBCT measurements, we could find reduction of the airway, and in the postoperative 6-month CBCT evaluation, the airway did not returned to the preoperative. However, in the postoperative 6-month CBCT evaluation, the patient did not complain about her snoring anymore (Fig. 3a). Another patient had narrower airway than her before surgery and in postoperative CBCT. However, she did not present snoring and any other airway compromises (Fig. 3b). Therefore, snoring did not always occur because of airway reduction due to orthognathic surgery [21]. There are several factors correlated with snoring such as head posture, respiratory habit, and tongue position [1, 3, 13, 16]. However, orthognathic surgery is not directly related to snoring and more research is needed.

There are many reports of airway reduction after orthognathic surgery [4], and some patients appear to have respiratory disorder or snoring [5, 12, 22]. If the surgeons understand the volume and width change of the airway between preoperative and postoperative, respiratory complications such as sleep apnea and snoring can be prevented [23]. Withal, if the evaluation of upper airway change in maxillary posterior impaction is possible, the surgeons would perform additional surgical procedures to prevent nasal airway problems [21].

There are many articles dealing with airway change after orthognathic surgery. Pereira-Filho VA et al. evaluated the correlation of maxillary advancement surgery and airway change. In this study, maxillary advancement surgery could dilate the volumes of nasopharynx and oropharynx but not hypopharynx [22]. R. Foltan et al. reported that maxillary advancement surgery could improve the respiratory condition of the upper airway [23]. But the relationship between maxillary posterior impaction surgery and upper airway change has not had been sufficient research. In the total volume of the airway, Lee Y et al. reported that bimaxillary orthognathic surgery for the correction of class III malocclusion affected the morphology by increasing the upper part and decreasing the lower part of the airway, but not the total volume [24]. However, Choi SK et al. reported that mandibular set back surgery was significantly associated with postoperative reduction of airway space [25]. In this study, we found that bimaxillary surgery involving maxillary posterior impaction could highly affect airway space of mandibular prognathism patients. So, the airway change due to bimaxillary surgery should be considered.

## Conclusions

The bimaxillary surgery involving maxillary posterior impaction can reduce the volume of airway of mandibular prognathism patients. Although total airway volume was reduced significantly, the changes in volume and diameter of nasopharynx were not statistically significant. The maxillary posterior impaction affects the nasopharyngeal airway, minimally.
